# Gambling in Victoria: Changes in Participation, Problem Gambling and Gambling Environment Between 2008 and 2018

**DOI:** 10.1007/s10899-024-10282-x

**Published:** 2024-02-05

**Authors:** Christine Anne Stone, Kristal Yeung, Lindsay Shaw, Rosa Billi

**Affiliations:** 1 Christine Stone Consulting, Melbourne, Victoria Australia; 2 Victorian Responsible Gambling Foundation, Melbourne, Victoria Australia

**Keywords:** Problem gambling, Prevalence, Gambling harm, Population study, Public health, Gambling ecosystem

## Abstract

Gambling and its impacts are an important public health issue. The relationship between gambling, problem gambling and gambling harm is complex and dynamic. Replicate prevalence studies are useful for surveillance and monitoring gambling impacts within jurisdictions. The purpose of this study was to compare changes in gambling and problem gambling in the Victorian adult population between 2008 and 2018 by investigating individual gambling activities and exploring their relationship with the Victorian gambling ecosystem. Gambling participation has decreased; problem gambling prevalence has not. Investigation beyond these summary measures reveals important details: (a) Electronic Gaming Machines (EGMs), casino table games, race and sports betting (‘high-risk activities’), informal private betting, and Keno, and their associations with problem gambling endure. Further, the strength of this association is unaffected by changes in product technology, delivery, or the Victorian environment in which they reside, (b) participation in high-risk activities, excluding EGMs, increased while for other activities they decreased, (c) EGMs continue to pose the greatest risk for Victorians, (d) males and young adults continue having a higher problem gambling prevalence rate and preferring both online gambling and high-risk activities (excluding racing favoured by an ageing, older cohort, and Keno, by all ages), and (e) gambling access and exposure proliferated enabling single site multiple gambling opportunities on high-risk activities. Young adults represented a new vulnerable group as they reach the legal gambling age. The most effective interventions (based on major falls in real expenditure (losses) on EGMs, the highest risk activity) were the smoking bans, removal of ATMs from venues and decreases in bet size. There is great potential for prevention, intervention, and minimising harm in the gambling environment.

## Introduction

Gambling and its impacts are an important public health issue. Estimates of the total burden to gamblers are not insignificant and equivalent to two-thirds the burden of *major depressive disorder* or *alcohol use and dependence*, and higher than *osteoarthritis* (Browne et al., 2016). In addition, gambling can harm the health and wellbeing of individuals, families, communities and society (Langham et al., 2015; Wardle et al., 2019, 2021). Gamblers have the potential to “export” about half the harms they experience to those around them (Li et al., 2017).

A key component of public health risk management is risk assessment including surveillance and monitoring (WHO, 2000). Population prevalence surveys are central in this regard. Jurisdictions surveying the population prevalence of gambling, problem gambling and correlates, usually` publish in the grey literature. Within-jurisdiction comparisons over time reveal fluctuations in gambling behaviours, the impact of a changing gambling ecosystem and the effectiveness of policies intended to minimise gambling harm.

In most countries, the majority of the adult population has gambled during their lifetime and more have gambled than not at some time over the past year (Calado & Griffiths, 2016). Comparisons of problem gambling prevalence across surveys can be problematic because of methodological differences. After standardisation of international studies between 1975 and 2012, Williams et al. (2012) estimated the adult population problem gambling prevalence to be in the order of 0.5–7.6% with lower values evident in the last decade. Consistent with this decline, a review of international studies between 2000 and 2015, found problem gambling prevalence varied between 0.1 and 5.8% (Calado & Griffiths, 2016).

The relationship between gambling participation and problem gambling prevalence is complex and dynamic. *The Conceptual Framework of Harmful Gambling* identifies antecedents of harm as both *gambling-specific* and *general factors* (Hilbrecht et al., 2020). Specific factors include gambling environments, exposure, products and resources, while cultural, psychological, social and biological are more general factors. The factors interrelate adding to the complexity. It follows that variations in factors over time have the potential to modify gambling harms experienced within a population. An environmental scan of the local ecosystem elaborates on some factors not captured in population prevalence studies.

The *gambling environment* is expanding. Globally, commercial gambling continues developing new markets and products, as they seek profit expansions. Governments and societies are often complicit in this growth as they pursue increased economic benefits from a profitable industry. Governments are responsible for legislation and monitoring and, in some jurisdictions, are the providers while simultaneously charged with the provision of resources delivering prevention and support/treatment services. One could say that they are gambling that benefits will outweigh harms. Local community acceptance and consumer demand also drive the system.

*Gambling exposure*, designed to attract and increase gambling opportunities, is ubiquitous. Associated with the 1980s gambling expansion (casinos, Electronic Gaming Machines or EGMs), participation and gambling problems increased, then plateaued or declined in many jurisdictions in the 2000s (Abbott, 2006; Wiebe & Volberg, 2007; Williams et al., 2012). The associated rapid growth in participation, gambling problems and harms, led to development of the availability or exposure hypothesis (Orford, 2005). The subsequent plateau and decline led to the adaptation hypothesis which predicts that in a mature market, problem gambling will eventually level out and decline as a consequence of decreased gambling participation (Abbott, 2006; Shaffer, 2005). While some argue these theories are alternatives, others propose they occur simultaneously as variations occur relating to gambling-specific factors and more general factors such as vulnerability of different population segments (Abbott et al., 2016).

Not all *gambling products* are equal. Certain structural characteristics (e.g., continuous forms) and contextual motivation characteristics (e.g. social or thrill-seeking), appear to be more often associated with harm than others (Hilbrecht et al., 2020) as is level of involvement (e.g. frequency of gambling and/or gambling on multiple activities) (Binde et al., 2017; Mazar et al., 2020; Yeung & Wraith, 2017). Associations vary with population segments, for example, males and females have different preferences concerning gambling products and intensity of gambling as do adults across the lifespan (Boldero et al., 2010; Hing et al., 2016). The level of risk from gambling products varies across jurisdictions moderated by the influence of local accessibility, and cultural, historical and regulatory factors (Calado & Griffiths, 2016).

The association between *gambling products* and problem gambling is not fixed but may rise and fall over time as features are modified (Binde et al., 2017). High-risk activities (EGMs, sports and race betting, casino table games) evolve with new features to attract individuals to gamble and to increase business profits. New technologies bring electronic forms of gambling into venues, potentially transforming low-risk activities into more continuous and rapid forms, and therefore riskier. The transition from land-based venues to myriad digital platforms removes geographic and temporal barriers to gambling activities whose origin (point of sale) may be anywhere in the world and limits the ability of jurisdictions to monitor activity-specific expenditure (losses) via local reporting.

Over the decade, the *Victorian gambling ecosystem* continued to change but not as dramatically as in the early 1980s. Real income from taxes and levies from gambling in 2018/19 was $1,800 million and real gambling expenditure^1^ (player losses) was $5,500 million and both have decreased from $2,000 million and $6,300 million in 2008/09 although recent racing and sports betting data are increasingly incomplete for Victorians since online betting became available from the Northern Territory (Queensland Government Statistician’s Office, 2021).

In Victoria, gambling is not one industry but multiple which intersect with various sectors of the population via different modes. Victorian legislation governs the land-based venues which serve a smorgasbord of gambling activities embedded within local neighbourhoods including major regional areas. Hotels and licensed clubs own and operate EGMs in dedicated areas of their premises, and offer Keno, race and sports betting, in addition to raffles, bingo and spinning wheels. Many have sports bars with racing and live sports on large screens and with Tabcorp face-to-face and electronic betting facilities. Tabcorp holding the exclusive wagering and betting licence in Victoria also operates nearly 100 standalone betting shops across the state. Victoria’s casino in the central business district offers access to EGMs, traditional table games, automated table games and poker tables. It also houses betting terminals identical to those at hotels and clubs. Similar gambling venues are available in all Australian jurisdictions, except for Western Australia where EGMs are only available at the casino.

Australian legislation, (*The Interactive Gambling Act 2001* (Cath)), limits the interactive gambling services that can be offered to Australians (Australian Government, 2023). The provision of internet casino gaming (such as poker, Blackjack and roulette) and online EGM gaming to Australians remains strictly prohibited however an exception is made for wagering in the form of race and sports betting, and lotteries. In 2008, gambling exposure was mainly via land-based venues. Since then, gambling online has increased markedly as in other jurisdictions for these permitted activities (Hing et al., 2021).

In 2016 we reported on gambling and problem gambling in Victoria, 2008 and the changes since 2003 (Abbott et al., 2016). Over that period, participation rates in all activities, individual activities and across most demographic groups, declined. However, problem gambling prevalence remained unchanged. Males and having lower education in both surveys, and younger age groups and metropolitan residents in the more recent survey, demonstrated higher risk profiles. Due to differences in survey methodologies, the problem gambling prevalence estimates were adjusted based on published recommendations (Stone et al., 2015; Williams et al., 2012).

Since 2012, few papers in peer-reviewed journals compare changes in adult population gambling and problem gambling within a jurisdiction. Similar to Victoria (Abbott et al., 2016), gambling participation decreased and with a decrease or unchanged problem gambling prevalence in Sweden between 1998 and 2009 (Abbott et al., 2014), the United States between 1999 and 2013 (Welte et al., 2015), New Zealand between 1985 and 2012 (Abbott, 2017) and Canada between 2002 and 2018 (Williams et al., 2021). Whereas gambling participation increased with an increase or no change in problem gambling prevalence in Iceland between 2005 and 2011 (Olason et al., 2015), Finland between 2007 and 2015 (Castrén et al., 2018), and Northern Cyprus between 2007 and 2018 (Çakıcı et al., 2021). A worldwide review of published national studies between 2000 and 2015 (Calado & Griffiths, 2016) mainly reported changes in problem gambling as do papers from Denmark between 2005 and 2010 (Ekholm et al., 2012) and 2005 and 2017 (Kragelund et al., 2022).

When considering the population risk of gambling harm, a summary measure such as trend in overall gambling participation may be too simplistic to explain variations, or lack thereof, in problem gambling prevalence. Given the strength of the association of problem gambling with factors such as high-risk gambling activities, intensity of gambling, and vulnerable population sectors, detailed examination of trends in these factors delivers a more meaningful risk assessment. Addition of relevant material from an environmental scan presents a more comprehensive overview than from the population surveys alone.

Since the 2008 survey (Hare, 2009), two large population gambling and health surveys have been conducted in Victoria (Hare, 2015; Rockloff et al., 2020) offering the perfect opportunity to further explore the local evolution of gambling and problem gambling. In all three, the PGSI was used to identify gambling problems and utilised a similar methodology obviating the need for adjustments to the problem gambling estimates. Harm was measured in 2014 and 2018, but the measures were sufficiently different that comparisons are not possible.

This paper aims to provide a comprehensive description of the relationship between gambling behaviour and problem gambling in 2018 and how they have changed since 2008 in Victoria, an Australian state with a population of 4.9 million adults in 2018. More specifically, the study investigated the changing dynamic between problem gambling and *gambling-specific factors* including gambling participation and intensity, gambling on individual activities and intensity, and online gambling modified by *general factors* of gender and adult lifespan. An additional aim was to expand the findings within the context of trends in the gambling ecosystem including policy developments, expenditure data, exposure and gambling types, and initiatives for harm prevention, reduction and protection.

## Methods

A secondary analysis was conducted on data from three large observational, cross-sectional population prevalence studies of gambling and health in Victoria: *A Study of Gambling in Victoria – problem gambling from a public health perspective (SGV) 2008 (*Billi et al., 2015; Hare, 2009*), Study of Gambling and Health in Victoria (SGHV) – Findings from the Victorian Prevalence Study 2014 (*Hare, 2015), and the *Victorian population gambling and health study (VPGHS) 2018–2019* (Rockloff et al., 2020).

*The SGV 2008* consisted of random digit dialling (RDD) of landline phones to conduct a CATI (Computer Assisted Telephone Interviews) survey of 15,000 Victorians, 18 years and older, in 2008. The sample was stratified by the eight state government regions and by high, medium, and low EGM expenditure in local government areas within these regions. High EGM expenditure areas were oversampled in the ratio of high 70%, medium 20% and low 10%. The response rate was 43.5% based on the more conservative calculation.

*The SGHV 2014* sampled Victorians aged 18 years and older using dual frame sampling using RDD methodology. The 13,554 CATI surveys conducted included 12,551 landline and 1,003 mobile surveys. Using the same method as the SGV, the sample was stratified by eight state government regions and by high, medium, and low EGM expenditure in local government areas. The response rates based on the more conservative calculation were reported as 58% for landlines and 31% for mobile surveys.

*The VPGHS 2018–2019* sampled 10,638 Victorians aged 18 years or above, using dual frame sampling split 50/50 between landline and mobile numbers and RDD methodology. While the distribution of Greater Melbourne/Rest of Victoria fell out naturally in the mobile sample, sampling quota was set in the landline sample (38% landline Greater Melbourne, 12% landline rest of Victoria, and 50% mobile). Unlike SGV and SGHV, there was no stratification in the VPGHS sample. The response rates for the landline and mobile frames were 6.0% and 12.6%, respectively.

### The Measures

Data on problem gambling, demographics, gambling participation and frequency over the previous 12 months were collected in the three studies for all respondents.

#### Problem Gambling

The PGSI was used to measure problem gambling. While the VPGHS 2018-19 used the original PGSI response options, the SGV 2008 and SGHV 2014 used the Queensland modification of the item response scale consisting of five points (never, rarely, sometimes, most of the time, almost always) (Queensland Treasury, 2001) rather than the original four-point scale (never, sometimes, often, almost always). Responses of ‘rarely’ and ‘sometimes’ were combined and given a score of 1. The range of scores remained from 0 to 27 as in the original PGSI. Cut-points for the total PGSI score were the same: 0, non-problem gambling (NPG); 1–2, low-risk gambling (LRG); 3–7, moderate-risk gambling (MRG); and ≥ 8, problem gambling (PG).

The main reason to use the modified scoring was to maintain the time series (Queensland Office of Regulatory Policy; Department of Justice and Attorney General, 2011). The Productivity Commission found the modified PGSI underestimated the number of PG and overestimated MRG based on simulations (Productivity Commission: Australia, 2010). However, an analysis of the two scoring systems used in a Victorian study found no significant differences in terms of problem gambling prevalence (Queensland Government, 2008).

As with many gambling studies, moderate risk gambling was combined with problem gambling into one group (MPG) in some of the analyses.

#### Gambling Participation

Gambling participation or yearly gambling refers to those who gambled at least once in a year; Monthly gambling to those who gambled at least 12 days in a year and Weekly gambling to those who gambled at least 52 days in a year. Regular gambling refers to monthly or weekly gambling. SGHV 2014 was not used for gambling frequency analysis due to the monthly and weekly gambling not being collected for all activities in the study. Gambling intensity refers to both gambling frequency and number of gambling activities participated.

#### Gambling Activities

The types of gambling activities asked between the three studies differed slightly, and those that overlapped in the three studies were analysed. Activities include private betting (informal playing cards at home for money), EGMs, table games (Blackjack, roulette, poker), racing (horse, harness or greyhounds race betting), sports betting, lotto (Lotto, Powerball or the Pools), Keno, scratch tickets, bingo and raffle tickets (raffles, sweeps and other competitions) and competitions (participation in SMS or phone-in competitions).

The questions for sports betting, and racing varied. In SGV 2008, event betting was included in the sports betting question resulting in a slight overestimation of sports betting. In *SGHV 2014* and *VPGHS 2018–2019* sports betting was asked as a stand-alone question. Both SGHV 2014 and VPGHS 2018-19 specified the inclusion of the Melbourne Cup in the racing question, while SGV 2008 did not. The modification was made based on findings from wave 3 of the *Victorian Longitudinal Study of Gambling & Health* (Billi et al., 2014). When asked specifically if they had placed a bet during the racing season (50 days), 6.1% of non-gamblers reported they had done so. Taking the under-reporting into account, we estimate that both population prevalence of gambling and race gambling in 2008 would increase by about 1.7% points. We are unable to extend this estimate to subgroup analysis. Adjusted values will be reported in brackets where possible.

Some gambling activities with low participation rates and/or not overlapping between the studies were excluded from the present study. These activities included speculative investment (3.2% of Victorian adults) in SGV 2008, eSports (0.4%) and fantasy sports (0.3%) in VPGHS 2018-19, and “other” gambling activities in all the studies (SGV 2008: 0.03%, SGHV 2014: 0.18% VPGHS 2018-19: 0.9%).

#### Gambling Mode (Derived from Gambling Channel)

Online gambling ever was identified by combining those who had endorsed “over the internet”, “computer games online”, and “on a mobile phone” for the SGV 2008, and “Australian-licensed bookmaker online or with a mobile app”, “overseas bookmaker online or with a mobile app”, and “online” for VPGHS 2018-19 as a gambling channel in at least one of the gambling activities they participated. SGHV 2014 was not used for online gambling analysis due to limited information collected for gambling channels in some gambling activities. Gambling by land-based venues only (LBVO) was identified as those who did not gamble online ever.

#### Age

Respondents were asked to provide their age in SGV and SGHV. If the exact age was not given, the interviewers would prompt age bands, and the midpoints of the age bands were imputed for analysis. If the exact age or the age band was not given, the missing age would be imputed with a random observation. In VPGH, fifteen age bands were used. The current study reports age in these categories for the three surveys: 18–24, 25–44, 45–64, 65–74, and 75+.

All expenditure is in Australian dollars.

### Analysis

Due to competing priorities for the SGHV 2014 survey, complete frequency data and gambling channels (mode of gambling) were not collected for all gambling activities. Results from data that were collected are reported in the tables however for simplicity mainly results from 2008 to 2018 are discussed.

Cross tabulations were generated to produce statistics for comparison between various subgroups. Weighted percentages with 95% confidence intervals were calculated. Overlapping of the 95% confidence intervals indicated no difference between estimates. The data was prepared for analysis using R programming with the “Base” package (R Core Team, 2020), and the “Tidy verse” package (Wickham et al., 2019). Weighted percentages and 95% confidence intervals were generated using the Package ‘srvyr’ (Freedman et al., 2021).

## Results

### Gambling Participation in 2018 and Changes Over Time

Victorians who gambled at least once over the previous 12 months (participation), at least monthly (monthly) and at least weekly (weekly) in 2008, 2014 and 2018, are reported by population, gender, age and activity in Table 1. Population gambling participation and intensity (frequency and multiple activities) continued to decrease from 2008 to 2018. Participation decreased slightly from 73 to 69% (adjusted value 74.7–69%). Larger reductions occurred in regular gambling: monthly from 41 to 29% and weekly from 23 to 14%. Gambling on multiple activities (3 or more) also decreased from 27 to 22%.

**Table 1:** Past year gambling participation and frequency, 2008, 2014 and 2018 by gender, age group and activity type

	2008			2014			2018		
	At least once	At least monthly	At least weekly	At least once	At least monthly	At least weekly	At least once	At least monthly	At least weekly
	% (95% CI)	% (95% CI)	% (95% CI)	% (95% CI)	% (95% CI)	% (95% CI)	% (95% CI)	% (95% CI)	% (95% CI)
Gender									
Male	72.6 (71.1, 74.1)	43.5 (41.9, 45.1)	25.0 (23.7, 26.3)	67.7 (63.8, 71.7)			69.7 (68.1, 71.3)	33.8 (32.2, 35.5)	17.3 (16.1, 18.6)
Female	72.5 (71.3, 73.7)	37.8 (36.5, 39.0)	20.4 (19.4, 21.4)	72.3 (69.5, 75.2)			68.2 (66.5, 69.8)	25.0 (23.6, 26.4)	11.5 (10.5, 12.5)
Age									
18–24	62.5 (58.7, 66.2)	21.7 (18.7, 24.9)	6.1 (4.6, 8.1)	58.3 (51.0, 65.6)			51.9 (48.2, 55.6)	13.8 (11.5, 16.5)	4.6 (3.3, 6.4)
25–44	71.3 (69.6, 72.9)	35.8 (34.1, 37.4)	15.9 (14.7, 17.2)	64.0 (59.6, 68.4)			65.9 (63.7, 68.0)	22.1 (20.3, 24.0)	7.3 (6.2, 8.6)
45–64	78.8 (77.4, 80.1)	50.0 (48.4, 51.6)	31.9 (30.4, 33.4)	76.0 (72.2, 79.8)			76.3 (74.4, 78.1)	36.2 (34.3, 38.3)	18.4 (16.9, 20.0)
65–74	75.5 (73.2, 77.7)	50.9 (48.2, 53.5)	34.1 (31.7, 36.7)	82.9 (78.7, 87.1)			78.0 (75.7, 80.3)	44.8 (42.0, 47.6)	28.9 (26.4, 31.5)
75+	65.3 (62.4, 68.3)	42.7 (39.7, 45.8)	30.1 (27.3, 33.1)	72.4 (66.0, 78.8)			66.4 (63.1, 69.7)	35.6 (32.4, 38.9)	23.5 (20.7, 26.5)
Activity									
Any activity	72.6 (71.6, 73.5)	40.5 (39.6, 41.5)	22.6 (21.8, 23.4)	70.1 (67.7, 72.5)			68.9 (67.8, 70.1)	29.3 (28.2, 30.3)	14.3 (13.6, 15.1)
Private betting	3.5 (3.0, 3.9)	1.0 (0.8, 1.2)	0.3 (0.2, 0.5)	2.8 (2.2, 3.5)			3.4 (3.0, 3.9)	0.6 (0.4, 0.8)	0.2 (0.2, 0.4)
EGM	21.5 (20.6, 22.3)	5.9 (5.4, 6.4)	1.7 (1.4, 1.9)	16.7 (14.8, 18.7)	4.6 (3.4, 5.7)	1.9 (0.9, 2.9)	14.1 (13.3, 14.9)	3.3 (2.9, 3.7)	1.1 (0.9, 1.4)
Table games	4.6 (4.1, 5.1)	0.4 (0.3, 0.6)	0.2 (0.1, 0.3)	5.1 (3.9, 6.2)	0.4 (0.2, 0.6)	0.2 (0.1, 0.4)	6.1 (5.5, 6.7)	0.4 (0.3, 0.6)	0.1 (0.1, 0.2)
Racing	16.4 (15.6, 17.2)	4.0 (3.6, 4.4)	2.1 (1.8, 2.4)	20.6 (18.8, 22.4)	3.9 (3.0, 4.8)	2.2 (1.5, 3.0)	19.8 (18.8, 20.8)	3.4 (3.0, 3.9)	2.0 (1.6, 2.4)
Sports betting	4.0 (3.5, 4.4)	1.6 (1.3, 1.9)	0.6 (0.5, 0.8)	4.8 (4.0, 5.6)	1.8 (1.3, 2.2)	0.8 (0.5, 1.1)	5.8 (5.2, 6.4)	1.7 (1.4, 2.1)	0.7 (0.5, 0.9)
Keno	2.3 (2.0, 2.6)	0.6 (0.5, 0.8)	0.4 (0.3, 0.5)	3.7 (2.6, 4.9)	1.3 (0.3, 2.2)	0.7 (0.1, 1.6)	3.3 (2.9, 3.7)	0.5 (0.4, 0.7)	0.1 (0.1, 0.2)
Lotto	47.5 (46.5, 48.5)	27.7 (26.8, 28.6)	18.3 (17.5, 19.0)	46.9 (44.4, 49.4)	24.9 (22.5, 27.4)	16.0 (13.9, 18.2)	44.3 (43.1, 45.5)	18.6 (17.7, 19.5)	10.8 (10.2, 11.5)
Scratch tickets	15.3 (14.6, 16.0)	3.9 (3.5, 4.2)	1.1 (0.9, 1.3)	10.7 (9.1, 12.4)			11.2 (10.4, 12.0)	1.6 (1.3, 1.9)	0.4 (0.3, 0.5)
Bingo	2.1 (1.8, 2.4)	1.1 (0.9, 1.3)	0.7 (0.6, 0.9)	2.6 (1.9, 3.3)			1.5 (1.2, 1.8)	0.6 (0.5, 0.8)	0.4 (0.3, 0.6)
Competitions	7.3 (6.8, 7.9)	1.2 (1.0, 1.5)	0.15 (0.1, 0.2)	5.8 (4.9, 6.7)			2.4 (2.1, 2.8)	0.4 (0.3, 0.6)	0.1 (0.1, 0.2)
Raffle tickets	42.9 (41.9, 43.9)	6.3 (5.8, 6.8)	1.2 (1.0, 1.4)	41.6 (39.2, 44.0)			37.3 (36.1, 38.4)	3.8 (3.4, 4.3)	0.7 (0.6, 1.0)

*Note* Sports betting in 2008 included events betting

Males and adults 45 years and older continued to gamble more intensely than females and younger age groups. Gambling participation did not differ between genders, and for both decreased slightly. While regular (monthly and weekly) gambling by both males and females decreased markedly, males continued to gamble more regularly. Regular gambling decreased across all ages except for adults aged 18–24 years whose weekly gambling remained unchanged. Participation was highest for adults aged 45–64 years and 65–74 years respectively in 2018, and has not altered in those aged 45 years and older, but has decreased in those under 45 years. Adults aged 45 years and older had the highest regular gambling prevalence.

More males than females continued to gamble on multiple activities, and both reported a significant decrease. Gambling on multiple activities was more common in adults aged 45–64 years and 65–74 years and lowest for adults aged 18–24 years and has decreased across all age groups.

Participation in gambling activities (Table 1).

In 2018, two activities persisted as the most popular: Lotto (44%) and raffle tickets (37%), followed by racing (20%), EGMs (14%) and scratch tickets (11%) and finally table games, sports, informal private betting, Keno, competitions and bingo. Participation trends varied across activities and increases occurred mainly with high-risk activities. Four activities increased: racing by 3.4 percentage points (adjusted value 1.7 percentage points), sports betting (by 1.8), table games (by 1.5) and Keno (by 0.9); informal private betting remained unchanged. Gambling on EGMs decreased by the largest margin, 7.4 percentage points. Decreases occurred with raffle tickets, competitions, Lotto, scratch tickets and bingo. Regular gambling (monthly and weekly) on informal private betting, table games, racing and sports, and Keno (monthly only), and competitions (weekly only) did not change. Monthly and weekly gambling decreased on EGMs, Lotto, scratch tickets, bingo and raffle tickets, and competitions (monthly only), and Keno (weekly only).

### Gambling Activities by Gender and Across the Lifespan

There are major differences in preferences for specific gambling activities by gender and across the lifespan and differences for 2008, 2014 and 2018 are detailed in Table 2.

**Table 2:** Gambling participation by gender and age group, for gambling activities, in 2008, 2014 and 2018

	Gender		Age group				
	Males	Females	18–24	25–44	45–64	65–74	75+
	% (95% CI)	% (95% CI)	% (95% CI)	% (95% CI)	% (95% CI)	% (95% CI)	% (95% CI)
Private betting							
2008	5.6 (4.8, 6.4)	1.4 (1.1, 1.8)	8.2 (6.3, 10.4)	4.4 (3.7, 5.3)	1.7 (1.3, 2.2)	1.0 (0.6, 1.6)	0.9 (0.4, 1.7)
2014	3.8 (3.0, 4.9)	1.9 (1.1, 2.8)	5.2 (3.1, 8.1)	3.9 (2.8, 5.4)	1.8 (1.1, 2.7)	0.7 (0.4, 1.2)	1.1 (0.6, 1.8)
2018	5.3 (4.5, 6.2)	1.5 (1.1, 2.0)	5.6 (4.1, 7.3)	4.9 (4.0, 5.9)	2.4 (1.8, 3.2)	1.0 (0.5, 1.6)	0.7 (0.4, 1.3)
EGMs							
2008	22.8 (21.5, 24.2)	20.1 (19.1, 21.2)	26.9 (23.7, 30.4)	16.6 (15.3, 17.9)	23.7 (22.3, 25.0)	24.7 (22.5, 27.0)	22.6 (20.1, 25.3)
2014	17.2 (14.6, 20.1)	16.3 (13.8, 19.0)	18.7 (13.7, 24.5)	12.7 (10.5, 15.2)	16.8 (14.0, 19.9)	21.6 (15.5, 28.7)	28.4 (15.2, 44.8)
2018	15.5 (14.3, 16.8)	12.7 (11.7, 13.8)	18.6 (15.9, 21.6)	12.0 (10.6, 13.5)	13.3 (12.0, 14.8)	16.7 (14.7, 18.8)	16.2 (13.8, 18.8)
Table games							
2008	7.4 (6.6, 8.4)	1.9 (1.5, 2.3)	12.9 (10.6, 15.6)	5.7 (4.9, 6.6)	2.1 (1.6, 2.6)	0.8 (0.4, 1.4)	0.2 (0.1, 0.6)
2014	7.5 (5.6, 9.9)	2.7 (1.9, 3.7)	11.2 (7.6, 15.6)	6.3 (4.9, 7.9)	2.0 (1.6, 2.5)	5.0 (1.0, 13.4)	0.4 (0.1, 0.9)
2018	9.1 (8.1, 10.2)	3.2 (2.6, 3.8)	15.7 (13.1, 18.4)	8.1 (7.0, 9.4)	3.3 (2.6, 4.2)	1.2 (0.6, 2.1)	0.5 (0.1, 1.2)
Racing							
2008	21.0 (19.7, 22.4)	12.0 (11.2, 12.8)	16.1 (13.2, 19.2)	19.6 (18.2, 21.0)	16.2 (15.0, 17.4)	11.3 (9.7, 13.1)	8.5 (6.7, 10.6)
2014	21.1 (18.7, 23.7)	20.2 (17.7, 22.9)	18.2 (13.2, 24.0)	19.4 (16.8, 22.2)	24.4 (21.0, 28.1)	19.2 (14.8, 24.1)	15.5 (11.8, 19.6)
2018	21.9 (20.5, 23.4)	17.7 (16.4, 19.1)	13.3 (10.9, 15.9)	20.6 (18.8, 22.4)	23.2 (21.4, 25.0)	19.2 (17.0, 21.5)	13.8 (11.5, 16.4)
Sports betting							
2008	6.5 (5.7, 7.4)	1.5 (1.2, 1.8)	6.9 (5.2, 8.9)	5.8 (5.0, 6.7)	2.4 (1.9, 3.0)	0.6 (0.3, 1.0)	0.6 (0.3, 1.1)
2014	8.0 (6.6, 9.6)	1.8 (1.1, 2.8)	9.2 (6.0, 13.2)	6.7 (5.1, 8.6)	3.1 (2.4, 3.8)	1.0 (0.6, 1.5)	1.1 (0.5, 2.0)
2018	9.7 (8.7, 10.8)	2.0 (1.5, 2.5)	11.5 (9.3, 13.9)	8.6 (7.4, 9.9)	3.4 (2.6, 4.2)	1.3 (0.8, 2.1)	0.6 (0.3, 1.1)
Keno							
2008	2.7 (2.2, 3.2)	2.0 (1.7, 2.4)	2.2 (1.3, 3.3)	2.1 (1.6, 2.6)	2.7 (2.3, 3.3)	2.3 (1.7, 3.2)	2.1 (1.3, 3.0)
2014	4.9 (3.0, 7.4)	2.7 (1.8, 3.8)	3.8 (1.8, 6.7)	2.8 (1.6, 4.4)	3.9 (2.3, 6.0)	6.1 (1.8, 13.9)	3.5 (2.3, 5.0)
2018	3.9 (3.3, 4.6)	2.6 (2.1, 3.3)	3.1 (2.0, 4.6)	3.1 (2.4, 4.0)	3.5 (2.7, 4.3)	3.8 (2.8, 5.0)	2.9 (1.9, 4.2)
Lotto							
2008	48.4 (46.8, 50.1)	46.6 (45.3, 47.9)	18.0 (15.3, 20.9)	47.4 (45.7, 49.1)	58.9 (57.3, 60.5)	53.5 (50.9, 56.1)	41.4 (38.4, 44.5)
2014	46.3 (42.6, 50.0)	47.5 (44.1, 50.9)	17.9 (12.7, 24.2)	40.4 (36.8, 44.2)	56.2 (52.2, 60.1)	67.7 (61.2, 73.8)	52.4 (42.1, 62.5)
2018	45.7 (44.0, 47.5)	42.9 (41.3, 44.6)	14.5 (12.0, 17.2)	39.5 (37.4, 41.7)	55.4 (53.3, 57.5)	59.7 (56.9, 62.4)	44.8 (41.4, 48.2)
Scratch tickets							
2008	13.3 (12.2, 14.4)	17.2 (16.3, 18.2)	17.4 (14.7, 20.3)	15.5 (14.3, 16.7)	15.2 (14.1, 16.3)	13.4 (11.7, 15.3)	14.1 (11.9, 16.5)
2014	8.1 (6.4, 10.1)	13.2 (10.6, 16.0)	15.9 (10.4, 22.7)	9.0 (7.4, 10.9)	9.7 (7.9, 11.7)	13.7 (6.7, 23.5)	8.7 (6.4, 11.4)
2018	9.2 (8.2, 10.2)	13.1 (12.0, 14.3)	12.2 (9.9, 14.7)	12.1 (10.7, 13.6)	9.9 (8.7, 11.2)	12.7 (10.8, 14.7)	8.9 (7.1, 10.9)
Bingo							
2008	0.8 (0.5, 1.1)	3.4 (3.0, 3.9)	2.4 (1.5, 3.7)	1.5 (1.2, 2.0)	1.6 (1.3, 2.0)	3.7 (2.8, 4.7)	4.6 (3.3, 6.1)
2014	1.4 (0.8, 2.3)	3.8 (2.7, 5.1)	2.8 (1.1, 5.8)	1.8 (1.2, 2.7)	3.2 (1.9, 4.9)	3.2 (1.4, 6.1)	3.2 (2.2, 4.6)
2018	0.5 (0.3, 0.8)	2.3 (1.9, 2.9)	0.7 (0.3, 1.5)	1.1 (0.7, 1.6)	1.6 (1.2, 2.2)	2.6 (1.8, 3.7)	1.9 (1.1, 2.9)
Competitions							
2008	4.9 (4.3, 5.7)	9.6 (8.9, 10.4)	6.0 (4.5, 7.7)	10.9 (9.9, 11.9)	6.8 (6.1, 7.6)	2.3 (1.6, 3.2)	0.7 (0.4, 1.2)
2014	4.1 (3.0, 5.4)	7.4 (6.1, 8.8)	5.5 (3.0, 9.1)	8.3 (6.5, 10.3)	4.6 (3.9, 5.5)	3.4 (1.7, 6.0)	1.4 (0.8, 2.3)
2018	1.3 (1.0, 1.7)	3.4 (2.8, 4.0)	1.0 (0.5, 1.8)	1.9 (1.4, 2.6)	3.2 (2.6, 4.0)	3.5 (2.5, 4.7)	1.7 (1.0, 2.7)
Raffle tickets							
2008	39.7 (38.1, 41.2)	46.0 (44.7, 47.2)	25.6 (22.5, 29.0)	43.0 (41.3, 44.7)	49.5 (47.9, 51.1)	45.5 (42.9, 48.1)	39.9 (36.9, 42.9)
2014	35.4 (32.2, 38.7)	47.5 (44.2, 50.9)	26.8 (20.5, 33.8)	38.0 (34.4, 41.6)	49.4 (45.6, 53.2)	48.8 (39.8, 57.9)	37.2 (29.3, 45.6)
2018	34.3 (32.6, 35.9)	40.1 (38.5, 41.8)	16.9 (14.3, 19.7)	35.0 (32.9, 37.1)	45.9 (43.8, 48.0)	43.7 (40.9, 46.5)	34.9 (31.7, 38.2)

*Note* Sports betting in 2008 included events betting

In all surveys around three times as many males gambled on table games, informal private betting and sports, and marginally more gambled on racing, EGMs and Keno. On the other hand, more females gambled on competitions, bingo, raffle and scratch tickets. Lotto tickets were bought by both equally. Since 2008 male participation increased in sports and Keno, with no change in informal private betting, table games, racing, Lotto and bingo. Female participation increased in racing and table games, with no change in informal private betting, sports and Keno, and decreased in lotto and bingo. Participation in EGMs, scratch tickets, competitions and raffle tickets decreased for both males and females.

Significantly more adults aged 18–24 years gambled on informal private betting, EGM, table games and sports; more aged 25–44 years gambled on informal private betting, table games, and sports; more aged 45–64 years gambled on racing, Lotto and raffle tickets; more aged 65–74 years gambled on EGMs, Lotto, bingo and raffle tickets. Participation in Keno and scratch tickets showed little differences across the lifespan.

Changes in participation in activities varied across the lifespan. Gambling increased significantly on races for adults 45 years and older and on Lotto for adults aged 65–74 years suggesting an age cohort effect, on table games for adults aged 25–44 and 45–64 years and on sports for adults younger than 45 years. Gambling decreased significantly on EGMs and Scratch tickets across all age groups, on Lotto for adults aged 25–44 years, on bingo for adults aged 18–24 years and 75 and older, on Competitions for adults younger than 65 years, and on raffle tickets mainly for adults younger than 45 years.

### Mode of Gambling - Online Gambling

The proportion of the adult population who ever gambled online was compared with those who used land-based venues only (LBVO) and the changes between the 2008 and 2018 surveys were examined. Gambling online has increased dramatically from 4.8 to 19% with a corresponding decrease in gambling in LBVO, from 69 to 49%. Online gambling was highest for Lotto (10%), races (6.8%) and sports (4.4%); a dramatic increase from 1.1% and less.

**Fig. 1:**
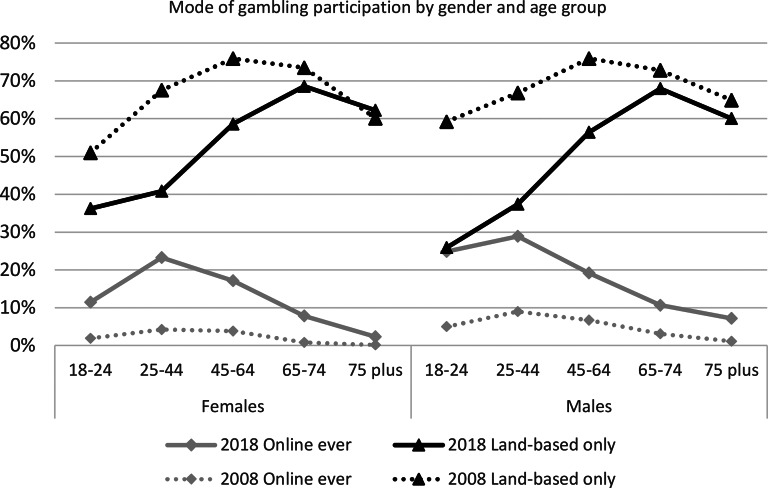
Mode of gambling participation by gender and age, 2008 & 2018

In 2018 a higher proportion of males, 22%, than females, 16%, gambled online, whereas a higher proportion of females, 51%, than males, 47% gambled in LBVO. Mode of gambling by gender and age-group, over time, are compared in Fig. 1. Gambling via LBVO predominated for males and females across the lifespan except males aged 18–24 years who gambled equally online and in LBVO. More males younger than 45 years gambled online than females and more females aged 18–24 years gambled via LBVO than males. Between surveys, LBVO gambling decreased for males and females aged 18–64 years and online gambling increased for both across the lifespan. In both surveys, the peak age for online gambling was 25–44 years, and for LBVO gambling shifted from 45–64 years to 65–74 years suggesting an age-cohort effect.

#### Race Gambling Online

Race gambling online has increased however gambling using LBVO predominated. Gambling on races using LBVO has decreased from 15 to 12% of the population. More males, 8.5%, than females, 5.1%, gambled online, whereas the gender difference was not significant for LBVO.

**Fig. 2:**
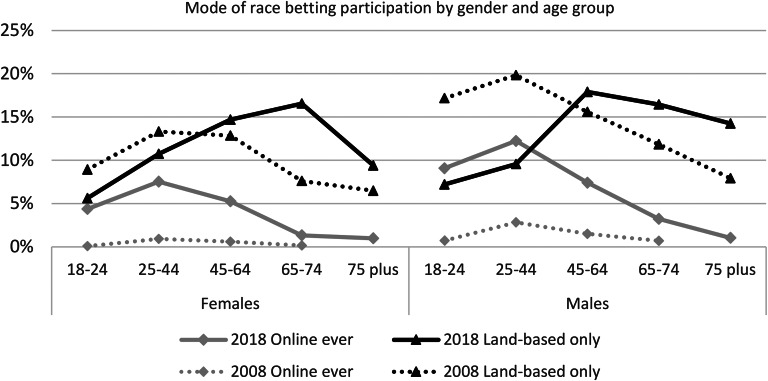
Mode of gambling on races by gender and age

Figure 2 compares the mode of gambling on races by gender and age over time. Gambling in LBVO predominated for males and females and across all age groups in 2008 and for males and females 45 years and older in 2018. LBVO gambling by males younger than 45 years has decreased and online increased by males and females under 65 years of age, suggesting some substitution of LBVO for online gambling. In both surveys, the peak age for gambling online was 25–44 years, but for LBVO gambling has shifted from 25–44 years to 45–74 years. Males aged 25–44 years (12%) gambled more often online than females (7.5%) of the same age whereas, for all other age groups, there was no significant difference in participation in online or LBVO modes. The figure demonstrates that the increase in race gambling was due to an increase in LBVO gambling by females aged 65–74 years and an increase in online gambling by males and females aged 45–64 years.

#### Sports Gambling Online

Gambling online on sports has increased so that in 2018 the online mode predominates. Three-quarters of the sports gamblers have gambled online, and the proportion reaches 90% for male sports gamblers aged 18–24 years.

**Fig. 3:**
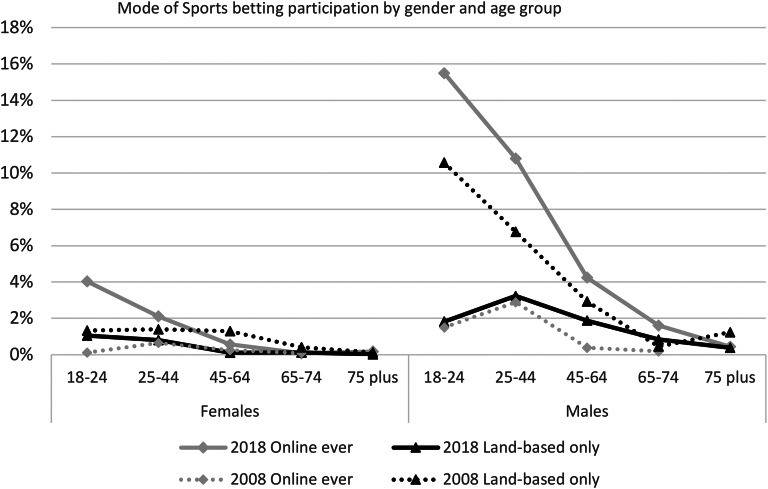
Mode of sports gambling by gender and age

Figure 3 compares the mode of sports gambling by gender and age for 2008 and 2018. The rise in online sports gambling was mainly driven by a large increase in young males and a smaller increase in young females gambling online. More males, 7.5%, gambled on sports online than females, 1.4% and this increased from 1.5% and 0.3% respectively. The peak age group for online sports gambling was 18–24 years at 10% (15% of males, 4.0% of females), a marked increase on the 0.8% in 2008, whereas the peak group in 2008 at 1.8% (2.9% males, 0.7% females) was aged 25–44 years. Young males aged 18–24 years have shown a major reduction in their usage of LBVO to gamble on sports from 11 to 1.8%. Online gambling on sports decreases with age.

### Problem Gambling Prevalence

The past year problem gambling prevalence in 2008, 2014 and 2018 for the Victorian adult population by age and gender are reported in Table 3. For some subgroup analyses the number of problem gamblers (PG) was small, therefore, to obtain robust results, the moderate risk and problem gamblers were combined to produce a “high-risk” group labelled MPG. The 2008 and 2014 results are described only when they differ significantly from 2018.

**Table 3:** Prevalence of problem gambling (PG) and high-risk gambling (MPG) by age and gender (2008, 2014 and 2018)

	2008		2014		2018	
	MPG	PG	MPG	PG	MPG	PG
	% (95% CI)	% (95% CI)	% (95% CI)	% (95% CI)	% (95% CI)	% (95% CI)
Adult population	3.1 (2.7,3.5)	0.7 (0.6,0.9)	3.6 (2.4,4.8)	0.8 (0.4,1.2)	3.1 (2.7,3.6)	0.7 (0.5,0.95)
Gender						
Male	3.9 (3.3,4.5)	1.0 (0.7,1.3)	5.2 (2.8,7.6)	1.0 (0.5,1.5)	4.4 (3.6,5.1)	1.0 (0.6,1.4)
Female	2.3 (1.9,2.7)	0.5 (0.3,0.7)	2.1 (1.3,2.9)	0.6 (0.0,1.3)	1.9 (1.5,2.4)	0.5 (0.2,0.7)
Age group						
18–24	4.9 (3.6,6.8)	0.6 (0.2,1.4)	3.5 (1.6,5.4)	0.6 (0.0,1.4)	5.8 (4.1,7.6)	0.5 (0.0,1.0)
25–44	3.0 (2.4,3.6)	0.9 (0.6,1.3)	3.2 (1.9,4.5)	1.2 (0.1,2.3)	3.2 (2.4,3.9)	0.9 (0.5,1.3)
45–64	3.1 (2.7,3.7)	0.8 (0.6,1.1)	2.4 (1.9,2.9)	0.7 (0.4,1.0)	2.8 (2.1,3.6)	0.9 (0.5,1.4)
65–74	2.1 (1.4,3.0)	0.2 (0.1,0.5)	8.1 (0.2,16)	0.3 (0,0.6)	2.3 (1.5,3.2)	0.5 (0.2,0.8)
75+	1.3 (0.7,2.4)	0.2 (0.1,1.0)	2.1 (1.2,3.0)	0.5 (0.0,1.0)	1.5 (0.7,2.4)	0.1 (0.0,0.2)

In 2018 the estimated past year PG prevalence was 0.7% based on a PGSI score of 8 and higher. An additional 2.4% were classified as moderate-risk gamblers (MRG). Low-risk gamblers (LRG) were estimated to be 6.7%, non-problem gamblers (NPG) 59% and non-gamblers (NG) 31%. The only changes were a small but significant decrease in the NPG and a corresponding increase in NG. These proportions correspond to over 36,000 PG, 118,000 MRG, over 329,000 LRG and approaching 2,912,000 NPG. There were 4,920,000 Victorian adults aged 18 and over (Rockloff et al., 2020).

In 2018, the link between PG and being male or younger age, and gambling more intensely, continued. Prevalence of PG, MRG and therefore MPG for males was at least double that for females. The MPG prevalence was highest in adults aged 18–24 years decreasing with age. Of those who gambled on multiple activities 2.6% were PG and 8.8% were MPG. In addition, of the adult males who gambled on multiple activities 3.4% were PG and 12% were MPG which was approximately double the prevalence for adult females, 1.7% PG and 5.6% MPG respectively.

Relationship between PGSI categories, gambling activities and the frequency of gambling.

The relationship between PG and MPG gambling and, the frequency of participation in any activity and in specific activities for 2008, 2014 and 2018 are reported in Tables 4 and 5. In 2018, PG and MPG prevalence was associated with gambling on specific activities and the frequency. Prevalence of PG was significantly higher in those who gambled on informal private betting, EGMs, Keno and bingo when compared with all gamblers; in regular monthly gamblers on EGMs and Keno than all monthly gamblers; and regular weekly gamblers on EGMs than in all weekly gamblers. MPG were associated with more gambling activities. The MPG prevalence was significantly higher in those who gambled on informal private betting, EGMs, table games, racing, sports, Keno and scratch tickets than all gamblers; in regular monthly gamblers on informal private betting, EGMs, table games, racing, sports and Keno than all monthly gamblers and regular weekly gamblers on informal private betting, EGMs and racing than all weekly gamblers.

**Table 4:** Prevalence of problem gambling (PG) among participants in major gambling forms at different frequency of gambling (2008, 2014 and 2018)

	2008			2014			2018		
PGSI8+	Yearly	Monthly	Weekly	Yearly	Monthly	Weekly	Yearly	Monthly	Weekly
	% (95% CI)	% (95% CI)	% (95% CI)	% (95% CI)	% (95% CI)	% (95% CI)	% (95% CI)	% (95% CI)	% (95% CI)
All gamblers	1.0 (0.8, 1.2)	1.7 (1.3, 2.1)	2.4 (1.8, 3.2)	1.4 (0.8, 2.3)	2.6 (1.5, 4.4)	3.6 (2.0, 6.6)	1.1 (0.7,1.4)	2.3 (1.6,3.0)	4.0 (2.7,5.3)
Private betting	1.3 (0.4, 3.9)	4.4 (1.4, 13.1)	3.8 (0.5, 22.6)	4.5 (2.1, 9.1)			4.6 (1.4,7.8)	4.2 (0.0,8.9)	6.6 (0.0,15.8)
EGM	3.0 (2.3, 3.9)	9.1 (6.8, 11.9)	16.5 (11.2, 23.6)	3.2 (2, 5)	10.6 (6.3, 17.2)	20.8 (9.7, 39.2)	3.6 (2.4,4.8)	12.4 (8.1,16.8)	28.3 (18.2,38.4)
Table games	3.8 (2.1, 6.9)	19.9 (8.4, 40.4)	34.1 (11.0, 68.4)	3.6 (1.5, 8.5)	29.8 (10.6, 60.4)	52.2 (19.8, 82.8)	2.6 (1.0,4.3)	16.4 (1.3,31.5)	
Racing	1.4 (0.9, 2.2)	3.4 (1.9, 6.0)	5.0 (2.5, 9.5)	2.1 (0.9, 4.8)	10.1 (4.1, 22.8)	16.2 (6.2, 36.3)	1.9 (1.2,2.7)	6.1 (2.8,9.4)	9.0 (3.6,14.4)
Sports betting	2.8 (1.4, 5.4)	4.7 (2.0, 10.7)	6.5 (1.8, 21.0)	7.5 (2.8, 18.5)	10.1 (4.3, 22.1)	12.8 (3.5, 37.2)	3.2 (1.1,5.2)	6.7 (1.6,11.8)	6.5 (0.0,14.0)
Keno	3.4 (1.7, 6.4)	5.8 (2.5, 12.6)	5.1 (1.6, 15.3)	3.1 (1.3, 6.9)	5.7 (1.9, 15.8)	4.9 (1, 21.3)	5.4 (2.0,8.7)	18.5 (3.3,33.7)	28.4 0.0,59.1)
lotto	1.1 (0.8, 1.5)	1.3 (0.9, 1.8)	1.4 (1.0, 2.1)	1.2 (0.6, 2.3)	1.2 (0.6, 2.2)	1.1 (0.5, 2.5)	1.2 (0.8,1.6)	1.9 (1.1,2.7)	2.0 (0.9,3.0)
Scratch tickets	1.7 (1.1, 2.4)	3.2 (1.9, 5.2)	4.9 (2.3, 10.1)	2.5 (1.3, 4.7)			1.9 (0.8,2.9)	4.3 (0.8,7.8)	10.3 (0.0,22.6)
Bingo	3 (1.7, 5.5)	3.7 (1.8, 7.6)	2.3 (0.7, 7.1)	3.5 (1.4, 8.4)			9.1 (2.5,15.8)	9.0 (0.3,17.7)	8.6 (0.0,19.3)
Competitions	1.2 (0.6, 2.7)	1.4 (0.4, 4.8)	10 (2.5, 32.7)	4.0 (1, 15.1)			1.5 (0.0,3.1)	3.0 (0.0,8.8)	10.4 (0.0,29.3)
Raffle tickets	0.7 (0.5, 1.0)	1.2 (0.6, 2.1)	1.4 (0.4, 5.0)	1.0 (0.4, 2.3)			0.8 (0.4,1.2)	1.5 (0.0,3.4)	4.8 (0.0,12.6)

*Note* Sports betting in 2008 included events betting

**Table 5:** Prevalence of high-risk gamblers (MPG) among participants in major gambling forms at different frequencies of gambling (2008, 2014 and 2018)

	2008			2014			2018		
PGSI3+	Yearly	Monthly	Weekly	Yearly	Monthly	Weekly	Yearly	Monthly	Weekly
	% (95% CI)	% (95% CI)	% (95% CI)	% (95% CI)	% (95% CI)	% (95% CI)	% (95% CI)	% (95% CI)	% (95% CI)
All gamblers	4.2 (3.7, 4.7)	6.6 (5.8, 7.5)	8.1 (7.0, 9.4)	5.9 (4.2, 8.3)	10.1 (6.9, 14.7)	11.6 (7.5, 17.5)	4.5 (3.9,5.1)	8.2 (6.9,9.4)	10.7 (8.7,12.7)
Private betting	13.1 (9.2, 18.3)	16.8 (9.6, 27.7)	12.8 (5.3, 27.5)	15.6 (9.3, 25.1)			15.4 (10.3,20.6)	29.2 (15.5,42.9)	36.5 (13.5,59.5)
EGM	11.5 (10.1, 13.0)	24.5 (21.1, 28.2)	35.1 (28.2, 42.8)	13.0 (8.7, 18.9)	36.7 (24.1, 51.4)	56.7 (31.9, 78.6)	12.5 (10.4,14.6)	29.4 (23.6,35.2)	46.5 (35.9,57.0)
Table games	16.3 (12.5, 20.9)	50.0 (33.3, 66.7)	62.1 (34.2, 84.5)	19.6 (9.0, 37.6)	42.8 (20.3, 68.7)	62.7 (28.9, 87.5)	11.6 (8.4,14.9)	43.0 (23.7,62.2)	46.0 (9.8,82.3)
Racing	7.2 (5.9, 8.7)	17.8 (14.0, 22.3)	22.7 (17.0, 29.4)	6.2 (4.5, 8.6)	20.6 (12.8, 31.4)	29.3 (16.5, 46.3)	6.7 (5.3,8.1)	18.7 (13.4,24.1)	22.7 (14.9,30.5)
Sports betting	13.1 (9.6, 17.6)	20.6 (13.9, 29.3)	27.1 (16.0, 42.1)	16.5 (10.3, 25.4)	29.2 (19.4, 41.5)	31.7 (17.3, 50.8)	13.3 (9.6,17.0)	19.6 (12.0,27.2)	20.8 (8.8,32.8)
Keno	11.0 (7.4, 16.1)	22.3 (12.7, 36.2)	28.3 (15.1, 46.8)	25.2 (11.2, 47.4)	45.3 (13.6, 81.3)	65.7 (22.3, 92.7)	13.6 (8.8,18.4)	28.7 (12.3,45.2)	36.5 (5.0,68.0)
lotto	4.7 (4.1, 5.4)	5.3 (4.5, 6.3)	5.0 (4.1, 6.1)	6.0 (3.9, 9.0)	7.9 (4.5, 13.6)	7.9 (4, 15)	4.6 (3.8,5.4)	6.0 (4.7,7.4)	6.4 (4.6,8.2)
Scratch tickets	6.1 (5.0, 7.4)	10.0 (7.5, 13.2)	10.1 (6.0, 16.5)	7.0 (4.7, 10.1)			7.8 (5.8,9.8)	15.4 (8.6,22.2)	21.2 (6.2,36.1)
Bingo	14.6 (10.0, 20.8)	16.6 (10.3, 25.7)	13.5 (8.0, 21.9)	7.5 (4.3, 12.8)			12.1 (5.0,19.1)	14.0 (4.3,23.8)	10.6 (0.0,21.4)
Competitions	4.3 (2.9, 6.4)	4.3 (1.9, 9.3)	15.9 (5.2, 39.3)	9.4 (4.7, 18.1)			5.3 (2.1,8.6)	11.5 (0.6,22.4)	14.1 (0.0,34.0)
Raffle Tickets	3.4 (2.9, 4.0)	4.4 (3.1, 6.2)	7.1 (3.2, 15.0)	4.5 (2.8, 7.4)			3.5 (2.7,4.2)	4.2 (1.4,7.0)	10.0 (0.0,20.2)

*Note* Sports betting in 2008 included events betting

In 2018, PG and MPG prevalence increased with frequency of gambling and were significantly higher in regular monthly gamblers and weekly gamblers than in all gamblers although the difference between weekly and monthly gamblers was not significant. When EGM gamblers were considered, the prevalence increased significantly with increasing frequency indicative of a “dose-response curve”. Over a quarter (28%) of weekly EGM gamblers were PG, compared to 12% of monthly and 3.6% of all EGM gamblers. Nearly half (46%) of weekly and 29% of monthly EGM gamblers were MPG compared to 13% of all EGM gamblers. A weaker relationship occurred with increasing frequency of gambling on table games (MPG only) and racing (both PG and MPG) although like all gamblers there was not a significant difference between monthly and weekly gamblers. In contrast, there was no increase in PG and MPG prevalence with increasing frequency of gambling on bingo, despite the high PG and MPG prevalence in bingo gamblers.

Problem gamblers often gamble on multiple activities which complicates the identification of the riskier activity. Analysis of gamblers and monthly gamblers on only one activity demonstrates that 2% of single-activity gamblers (SAG) and 3% of single-activity monthly gamblers (SAMG) are MPG. Concerning individual activities, 15% of SAG-EGM and 41% of SAMG-EGM are MPGs; 10% of SAG-table games and 61% of SAMG-table games; 2% of SAG-Racing and 8% of SAMG-racing; 3% of SAG-Sports and 18% of SAMG sports are MPGs. There were no monthly keno-only gamblers. Findings further support the higher risk from EGM and Table games, and monthly racing and Sports.

### MPG and Online Betting

The PG prevalence and MPG for adults who gambled on all activities, Lotto, races and sports by mode of gambling (ever online and LBVO), and by gender and age group were analysed and compared between 2008 and 2018. Lotto results were very similar to all gambling so are not described further. Only the 2018 results for MPG by mode of gambling and by gender and age-group are reported in Table 6.

**Table 6:** Prevalence of “High-risk” gamblers (MPG) by mode of gambling (“online ever” or “land-based venues only”) by gender and age, 2018

	Total	Sports	Race	Lotto
	% (95% CI)	% (95% CI)	% (95% CI)	% (95% CI)
All gamblers	4.5 (3.9,5.1)	13.3 (9.6,17.0)	6.7 (5.3,8.1)	4.6 (3.8,5.4)
Mode: online ever				
All	7.2 (5.7,8.8)	12.6 (8.4,16.7)	8.1 (5.5,10.8)	5.3 (3.4,7.1)
Gender				
Female	4.6 (2.8,6.5)	4.2 (0.0,10.3)	3.5 (1.0,6.0)	4.9 (2.3,7.5)
Male	9.3 (6.9,11.6)	14.2 (9.4,19.0)	11.1 (7.1,15.1)	5.6 (3.0,8.2)
Age group				
18–24	17.5 (11.2,23.8)	21.7 (12.2,31.2)	19.8 (9.2,30.4)	13.0 (2.0,24.1)
25–44	6.7 (4.5,8.9)	8.1 (3.1,13.0)	6.4 (2.9,9.9)	5.6 (2.9,8.4)
45–64	5.6 (3.1,8.0)	13.4 (2.7,24.2)	6.9 (2.1,11.8)	3.9 (1.2,6.7)
65–74	2.8 (0.5,5.0)	13.1 (0.0,32.7)	8.6 (0.1,17.1)	3.0 (0.6,5.4)
75+	0.6 (0.0,1.7)		2.5 (0.0,7.4)	1.2 (0.0,3.6)
Mode: land-based venues only				
All	3.5 (2.9,4.1)	14.6 (6.4,22.9)	6.2 (4.5,7.9)	4.4 (3.6,5.3)
Gender				
Female	2.3 (1.6,3.0)	22.1 (0.5,43.6)	3.3 (1.5,5.0)	2.5 (1.7,3.4)
Male	4.9 (3.8,6.0)	13.0 (4.1,21.9)	9.0 (6.2,11.9)	6.3 (4.8,7.8)
Age group				
18–24	8.0 (4.3,11.8)	16.3 (0.0,39.0)	14.4 (4.1,24.7)	12.1 (4.3,20.0)
25–44	3.5 (2.2,4.7)	14.7 (3.9,25.4)	4.3 (1.7,6.9)	5.2 (3.3,7.2)
45–64	3.2 (2.2,4.2)	15.1 (0.0,34.0)	6.8 (3.9,9.7)	4.1 (2.8,5.4)
65–74	2.9 (1.8,4.1)	10.9 (0.0,27.1)	5.8 (2.0,9.5)	3.4 (1.9,4.8)
75+	2.5 (1.1,3.8)		5.1 (0.8,9.3)	3.0 (1.2,4.8)

Male gamblers reported twice the MPG prevalence compared with female gamblers whether they were online or LBVO gamblers. The MPG prevalence among online and LBVO gamblers was highest in adults aged 18 to 24 years and decreased with age.

The MPG prevalence was not significantly different among those who gambled on races online or LBVO. Males who gambled on races online or in LBVO reported three times the MPG prevalence compared with females online or in LBVO. There was some indication that the MPG prevalence was highest in adults aged 18 to 24 years, 20% online gamblers and 14% LBVO gamblers, and decreased with age although because of wide confidence intervals, these results were not significant.

MPG prevalence was not significantly different between those who gambled on sports online or LBVO. Males who bet on sports online reported a higher MPG prevalence compared with females however this difference was only approaching significance due to wide confidence intervals. There was some indication that the MPG prevalence at 22% was highest in adult online sports gamblers aged 18 to 24 years, and again because of wide confidence intervals these results were not significant. The breakdown of LBVO sports betting by gender and age was limited by the small proportion using this mode to gamble on sports.

## Discussion

Problem gambling prevalence in the adult population in Victoria has not changed despite a decrease in participation and gambling intensity. In 2018, 69% gambled at least once over the previous 12 months, 29% gambled at least monthly and 14% at least weekly. Over the decade, gambling participation decreased slightly and gambling intensity decreased further by 5–10 percentage points. These findings are promising given the association of problem gambling with gambling intensity. However, in the US the decrease in frequent gambling was accompanied by an increase in the size of bets (Welte et al., 2015) and in Canada by the total expenditure (Williams et al., 2021) raising the possibility that more of the burden is borne by a smaller section of the population. In 2018, the estimated problem gambling (PG) prevalence (0.7%), moderate-risk gambling (MRG), (2.4%) and low-risk gambling (LRG), (6.7%) have not changed significantly. These trends continue those reported previously between 2003 and 2008 in Victoria (Abbott et al., 2016).

Few jurisdictions have compared the findings of replicate prevalence studies in the published literature over the same period. Consistent with our findings, and continuing trends previously reported (Williams et al., 2012), most studies describe falling participation with either stable or decreasing problem gambling prevalence. Six have reported a decrease in gambling and intensity: three with no change in problem gambling prevalence: Sweden (Abbott et al., 2014), the United States (Welte et al., 2015) and (Abbott, 2017) and three with a decline in problem gambling: Hong Kong and Singapore (Calado & Griffiths, 2016) and Canada (Williams et al., 2021). Britain differed depending on which problem gambling measure was compared. Gambling decreased between 1999 and 2007 then increased to its previous level in 2010. Problem gambling as measured by the PGSI did not change between 2007 and 2010 whereas using the DSM-IV, the 2010 level was significantly higher than the 1999 and 2007 levels. Other studies report rising participation rates with no change in problem gambling in Finland (Castrén et al., 2018) and France (Calado & Griffiths, 2016) or a rise in problem gambling in Iceland (Olason et al., 2015), and in North Cyprus (Çakıcı et al., 2021).

Our study shows the value of further investigation of individual gambling activities in understanding problem gambling trends. Summary measures conceal why, despite decreases in participation and gambling intensity; problem gambling prevalence remained unchanged. Some gambling activities have stronger association with problem gambling and gambling activities with higher participation rates influence the overall problem gambling prevalence more than those with lower participation rates. Furthermore, considering problem gambling prevalence is low and large studies are necessary to show variations in prevalence, the higher gambling prevalence presents a more sensitive indicator of developments that can potentially affect problem gambling.

This study demonstrates that EGMs, casino table games, race betting and sports betting continued to be high-risk activities in the Victorian population using a variety of criteria: Gamblers on these activities have a higher PG and MPG prevalence; presence of a dose-response curve with increasing frequency and a higher MPG prevalence in single activity gamblers or monthly gamblers. Those who gambled on informal private betting and/or Keno and experienced problems with gambling also gambled on multiple activities. Analysis of pooled Australian state prevalence studies demonstrated that EGM was the major contributor to the problem gambling burden followed by casino games, race and sports betting (Browne et al., 2023). Thus, confirming these four activities are high-risk activities across Australia. Worldwide, gambling-related factors such as EGM and Internet gambling were shown to have the largest effect size followed by casino gambling, poker, and daily lotteries (Allami et al., 2021). Using both cross-sectional and longitudinal study designs, Williams et al. (2022) confirmed the importance of EGM gambling on both current and future problem gambling.

While overall gambling participation decreases were observed over ten years in Victoria, these were associated with activities that had high rates of participation at the population level yet posed lower risk overall (e.g., Lotto 44.4%, raffles 37.4%). Two high-risk activities were next. Race betting increased from 2008 to 2018 (16.4 to 19.8% - adjusted increase was still 1.7% points) whereas EGMs decreased (21.5 to 14.1%). Participation rates increased in the other high-risk activities, sports and table games that have relatively lower participation at the population level, and therefore did not reflect in the overall gambling participation. As such, the decline in the overall participation did not result in a concomitant PG prevalence decline. To reduce the PG prevalence and attendant harm, participation in high-risk gambling must drop.

The association between forms of gambling and PG are not necessarily fixed over time (Binde et al., 2017). Another hypothesis posits that a modification in risk of some activities has occurred. Factors that can affect the risks associated with specific activities include rapid increase in online and venue access plus modifications of structural characteristics. MPG prevalence associated with each activity has not varied significantly since 2008, suggesting that developments relating to activities have not yet affected their potential risk for problem gambling, although they may in the future. There is potential for reduction in the structural characteristics that contribute to the riskiness of these products.

Online gambling (followed by EGM) has been demonstrated to have the strongest associations with problem gambling worldwide (Allami et al., 2021) and is not only associated with problem gambling but has been recognised as a separate disorder: *Gambling disorder, predominately online* (World Health Organization, 2018). The dramatic increase in participation in online gambling evident in Victoria has been reported elsewhere (Abbott, 2017; Abbott et al., 2014; Çakıcı et al., 2021; Castrén et al., 2018; Pallesen et al., 2021; Welte et al., 2015; Williams et al., 2021). In Victoria, participation was more common among males and younger age groups although LBVO gambling still predominated for both males and females across all ages except males aged 18–24 years. MPG Prevalence among online gamblers was 7.2%, and higher for males 9.3% and the youngest age group 17.5%. It was higher again in those who gambled online on sports or racing; for example, one in five males aged 18 to 24 years who gambled on sports or racing were MPG. Of note, in Victoria in 2018 sports betting was the first gambling activity to be accessed predominantly online with only 25% of sports bettors in general and 10% of male sports bettors aged 18–24 years gambling in LBVO. The Victorian MPG prevalence in online gamblers is at the lower end of the range worldwide of 2.7–26.2%, is consistent with the male predominance but younger than the average age group reported in a review of studies of online gambling. However, as the authors note comparison between studies is difficult due to the different years of study and associated rapid adoption of technology, definitions of online gambling, measures of gambling disorder, locations and population differences (Mora-Salgueiro et al., 2021).

In Victoria, the risk of problem gambling varies within a population. Aggregated population information hides major differences in gambling and problem gambling across population segments. In 2018, the male PG and MPG prevalence continued to be twice that of females. Gambling participation decreased slightly, and gambling intensity declined further for both genders. However, males continued to gamble more intensely and on riskier activities: slightly more on EGMs, race betting, and Keno but three times as much on informal private betting, sports betting and table games. In contrast, females showed preferences for less harmful activities. Volberg (2003) reported on the feminisation observed with EGM gambling. Our study found some feminisation with race gambling but not EGMs over the decade. In 2018, race participation returned to the 2003 levels (Abbott et al., 2016; The Centre for Gambling Research: Australian National University, 2004) however in both cases, male race gamblers still predominated.

The MPG prevalence was highest among the youngest age group of 18–24 years, decreased with age and this has not changed over time. Yet young adults in Victoria gamble less, and less intensely than their older counterparts indicating that other factors are driving the problem gambling in this group. Choice of activity is one factor. More adults aged 18–24 years showed a preference for the high-risk activities of informal private betting, table games, sports, and EGMs than others across the lifespan. EGM gambling across all ages decreased, particularly for those aged 45–75 years. Racing was an exception and was preferred by an ageing cohort, adults aged 25–44 years in 2008 and aged 45–64 years in 2018. Adults older than 45 years showed a preference for high participation (e.g., raffles, Lotto) and lower-risk activities.

Few studies have compared changes in gambling by age group. Like Victoria, comparison studies in Sweden and the US found that despite the youngest age group having the lowest participation level and gambling frequency, they have the highest problem gambling level. Participation across age groups decreased especially those aged under 25 years in Sweden between 1998 and 2009, and in the US between 1999 and 2013, consistent with the decrease in Victoria over a similar period (Abbott et al., 2014, 2016; Welte et al., 2015). In contrast, participation increased across all ages in Iceland (Olason et al., 2015).

A profile of decreasing risk of problem gambling across the lifespan is consistent with elements of both accessibility and adaptation occurring within populations as Abbott et al. (2016) propose. The high problem gambling prevalence in young adults, 18–24 years, is indicative of the vulnerability of a naïve sector of the population as they reach the legal age of gambling. It happens despite or maybe because of some adolescent engagement with these products before the legal age is reached (Boldero et al., 2010) and the possible convergence of gaming and gambling (Dussault et al., 2017; Hayer et al., 2018; Zendle & Cairns, 2019). PG is not a permanent state nevertheless the impact/legacy of experiencing problem gambling at such an important life stage is potentially lifelong. The decrease in PG as each cohort ages is consistent with adaptation.

### The Victorian Gambling Environment and Changes in Participation

Changes in gambling participation do not occur in a vacuum. Governments influence by regulating access and availability, and provision of prevention, intervention, treatment and support services. In Victoria, the growth in gambling on casino table games, racing, sports and Keno can be linked with expansions in these forms and an increase in losses in real terms.

Gambling on EGMs and casino table games persist in their strong association with problem gambling in Victoria. Although EGM gambling and gambling losses are decreasing and EGM numbers have been capped at 30,000 since 1997 (considering population growth, effectively reduces the number per adult over time), Victorians lose more money on EGMs than any other gambling activity. In 2018-19, total real gambling losses in Victoria were $5.46 billion, with $2.7 billion, or nearly 50%, coming from EGMs at hotels and clubs. When losses on EGMs and table games in the casino are included, the figure reaches $4.37 billion or 80% of all gambling losses in Victoria (Queensland Government Statistician’s Office, 2021). Note that losses in the casino for EGMs separate from table games are unavailable for the period covered in this paper.

Over the decade various interventions have been introduced to reduce gambling harm in clubs and hotels where most (26,500) of the EGMs are housed. Venues have been required to have a Responsible Gambling Code of Conduct (RGCode) and a Self-Exclusion Program (SEP) (“*Gambling Legislation Amendment (Problem Gambling and Other Measures) Act 2007* (Vic),“) and offer a pre-commitment scheme directed to individuals (“*Gambling Regulation Amendment (Pre-commitment) Act 2014* (Vic),“). Both SEP and pre-commitment are voluntary for gamblers to use. These interventions probably had a small effect however there is little evidence for interventions that are voluntary in nature. Major reductions in real EGM losses year on year are associated with broader regulatory interventions such as the introduction of smoking bans (2002), a combination of RGCode and SEP being introduced, a halving of bet size to $5 (new machines in 2008 and all machines 2010) and Automatic Teller Machines (ATM) removal (2012) (Lal & Siahpush, 2008; Livingstone et al., 2014, 2019; Thomas et al., 2013). The recent limits to EFTPOS withdrawals at clubs and hotels with gamblers being required to interact with staff to initiate withdrawals (2018) have yet to be evaluated.

Casino EGMs have higher potential for harm than those in clubs and hotels based on their structural characteristics. Their maximum bet per spin of $10 continues. In 2014, several changes were allowed (“*Casino and Gambling Legislation Amendment Act 2014* (Vic),“). EGM numbers increased from 2,500 to 2,628 of which a maximum of 1,000 can operate in “unrestricted” mode. Originally in limited “high roller” areas, they were then made available on any machine at the casino, provided no more than 1,000 EGMs are in “unrestricted” mode at any one time. EGMs operating in “unrestricted” mode have no minimum spin rate, no maximum bet limit per spin, no load-up limit, and can operate in Auto Play (the gambler does not need to press the button for each bet).

Gambling on table games is only available in the casino and has increased possibly associated with their expansion over the decade and minimal effective interventions. Poker tables increased from 50 to 100 in 2009 and gaming tables increased from 350 to 400 in 2009 then to 440 in 2014, and Fully Automated Table Game (FATG) terminals (approximately 10 per table) from 0 to 200 in 2009 and 200 to 250 in 2014 (“*Casino and Gambling Legislation Amendment Act 2014* (Vic),” ; Victorian Commission for Gambling and Liquor Regulation (VCGLR), 2009). Harm minimisation initiatives mainly of voluntary nature introduced over the ten years include a state-wide voluntary pre-commitment scheme, allowing gamblers to set time and money limits, however, the very high limits permissible curb its effectiveness. The total impact of the 2014 changes at the casino (EGMs and table games) was an 18% increase in real losses in 2014-15 suggesting that the harm arising from gambling in the casino is increasing (Queensland Government Statistician’s Office, 2021).

In Victoria gambling on racing and sports betting is the next major source of gambling losses and is associated with gambling harm (Queensland Government Statistician’s Office, 2021). Gambling on both has increased over the decade however they are preferred by different sectors of the population: racing by an older and aging cohort and sports by males in the younger age groups. Note that around 75% of sports gamblers gambled online in 2018. Losses on racing have decreased in real terms and on sports reached a peak in 2017/18. Unfortunately, recent data for gambling by Victorians is incomplete as gambling transitions to online and losses have been attributed to the Northern Territory, a major supplier of online wagering.

Since 2008 wagering (gambling on racing, football, trackside and sports) opportunities expanded with the new licensing of TAB (now Tabcorp) for retail and online betting, and removal of restrictions on interstate online bookmakers after a high court case (“*Betfair Pty Ltd and Matthew Edward Erceg V State Of Western Australia*,” 2008). Supply based on the number of races and race meetings conducted in Victoria increased, mainly due to greyhound racing rather than thoroughbred or harness racing which were unchanged in numbers (Greyhound Racing Victoria, 2013, 2014, 2016 & 2019; Harness Racing Victoria, 2013 & 2019; Racing Australia Limited, 2019). Over the decade, various online bookmakers developed relationships with the Australian Football League (AFL), the dominant Victorian football league, then with some individual AFL clubs and finally, the official AFL broadcast station further extending their marketing opportunities (McClure, 2022). By 2017, AFL Victoria and all 10 Victorian clubs had signed up to the “Love the game not the odds”, a program promoting sports betting-sponsorship-free clubs (Victorian Responsible Gambling Foundation, 2023). Australian wagering advertising grew significantly from $89.7 million to $254.1 million during the seven years from 2011 to 2018 (Hetherington & Phillips, 2023). At the national and state level, restrictions were gradually introduced including limits around times when match odds could be advertised during broadcasts, then all gambling advertisements during certain hours of broadcasts, and ad bans near schools, roads and public transport (Australian Communication and Media Authority (ACMA), 2023; “ Gambling Legislation Amendment Act 2018 (Vic),“).

Keno gambling has grown associated with the increased accessibility to more venues, from the 500 clubs and hotels that offered EGMs, to over 600 clubs and hotels, as well as nearly 100 standalone betting outlets in 2012 (Victorian Commission for Gambling and Liquor Regulation (VCGLR), 2013). Real losses on Keno virtually doubled in the following year as a result (Queensland Government Statistician’s Office, 2021). The addition of TAB outlets to clubs and hotels brings additional access to gambling on racing and sports to a venue that also serves alcohol.

All published replicate prevalence studies excluding Iceland mention an expansion of the gambling industry and gambling legalisation over the period covered by their reports. Some detail specific developments relating to individual gambling activities. All report an increase in online gambling associated with increased availability of this form of access. Similar to Victoria, a decrease in EGM gambling has been noted in NZ, Sweden (but not past 30 days), Finland, Iceland and Canada, with no change in the US (Abbott, 2017; Abbott et al., 2014; Castrén et al., 2018; Olason et al., 2015; Welte et al., 2015; Williams et al., 2021).

The trends in gambling participation differ across countries, and in some cases, participation decreased despite the expansion and promotion of specific gambling activities. In areas where access to casinos expanded, casino gambling increased in Canada and North Cyprus whereas there was no change in the US and a decrease in New Zealand, Sweden and Finland. With the state-owned poker website and an increase in EGMs, Sweden experienced an increase in poker participation and problem gambling in young men, and an increase in EGM gambling. Despite promotion and marketing of new ways of race betting, it declined in Canada, Sweden, and New Zealand (Abbott, 2017; Abbott et al., 2014; Çakıcı et al., 2021; Olason et al., 2015; Welte et al., 2015; Williams et al., 2021).

Problem gambling prevalence has not changed in Victoria since 2003. Of major concern is whether it will rise because of the major social and economic shocks experienced during the Covid pandemic. There are early indications that this may happen. After dropping to a low of $1,565 million in 2020/21 during lockdowns, Victorian EGM expenditure (losses) in hotels and clubs has already risen to a similar amount of $1,580 million in the first half of 2022/23 (Victorian Gambling and Casino Control Commission, 2023). Two replicate studies reported the impacts of major social and economic shocks demonstrating important cultural and social factors that influence gambling behaviour and problem gambling. An increase in participation in Iceland was accompanied by an increase in problem gambling post the severe global financial crisis (Olason et al., 2015) Pathological gambling doubled in North Cyprus associated with an exponential increase in the gambling industry when Turkey banned gambling in 1997 as well as major immigrant influx (Çakıcı et al., 2021). Worldwide, initial impacts of the Covid lockdowns were mainly decreasing gambling with varying transitions to online gambling. Increases in gambling or problem gambling were associated with already vulnerable groups. Post lockdown there have been some reports of an increase in gambling. (Brodeur et al., 2021; Hodgins & Stevens, 2021).

### Strengths and Limitations

This is the first Australian study to compare the changing risk profiles over two multi-year periods. While between 2003 and 2008 we reported gambling on all activities decreased; in particular, EGMs (by 36%), racing (by 42%), table games (by 37%) and sports betting (by 28%) (Abbott et al., 2016). Between 2008 and 2018, we report continued decreases in EGMs (by 34%) but increases in race betting (by 21%), table games (by 32%) and sports betting (by 45%). Although both periods demonstrate no change in problem gambling prevalence, the increase in high-risk activities may portend an increase.

A strength of this study is that comparisons are between three large representative population-based surveys. The study designs, and sampling and weightings techniques were carefully optimised according to recognised methodologies allowing a robust comparison of changes over 10 years. The large computer-assisted telephone interview (CATI) surveys were all described as gambling and health surveys (Williams & Volberg, 2009). Gambling behaviour about all gambling activities was asked of all participants and the PGSI was administered to all gamblers. Thus, obviating the need for adjustments for the many methodological differences between the survey necessary for the previous paper on changes in Victoria (Abbott et al., 2016; Stone et al., 2015; Williams et al., 2012).

Worldwide, few studies in the 2000s have compared prevalence studies changes over time and even fewer have described in any detail the gambling ecosystem. The comparison of population-based studies on the same population over a decade provided valuable insights not only into gambling and problem gambling but also the influential factors within the gambling environment, regulation and policy. We have sought to triangulate the changes over time derived from the CATI-based prevalence studies with variations over the same period in state and nationally reported expenditure (gambling losses) data, industry developments, government regulation and legislation, and major interventions. Furthermore, comparisons have been made with the few replicate studies that have been published in the peer-reviewed literature.

As with many telephone surveys over this period, a limitation is that sampling has transitioned from 100% landline or household survey to a mixed landline and mobile phone or personal survey to sample a broad section of the population. In addition, in 2008 and 2014 but not in 2018 the sample was enriched for problem gamblers by oversampling high EGM spend areas. Other variations were that the 2008 and 2014 surveys used the Queensland modification of the item response scale for the PGSI whereas in 2018 there was a return to the original 4-point scale (Ferris & Wynne, 2001) and finally there was a modification to the racing question to capture the self-reported non-gamblers who had gambled on the Melbourne Cup, although the impact was deemed small.

The response rates were not consistent between the studies, with reductions observed over time. The response rates between SGV 2008 and SGHV 2014 can be compared directly since the two studies used similar methods. It is not the case for VPGHS 2018-19 as a different method was used to conduct the survey and to calculate the response rate. Despite the differences, the reduction in the response rate observed is consistent with the global decline trend in telephone survey response rates (Lavrakas et al., 2017; Rockloff et al., 2020).

## Conclusion

Monitoring and surveillance are essential to understand developments over time and offer insights into the potential to reduce harm in the population. Population participation rates are a summary measure and may be more useful as an indicator of acceptance of gambling in the community. Concerning Victoria and the changes over ten years, participation and involvement decreased however the problem gambling prevalence did not. Participation decreased in low-risk products. An increase in gambling on three of the four high-risk gambling products was detected within the context of a still-expanding but mature market. The association of these products with problem gambling has not changed. Despite the decrease in EGM gambling, they remain the highest risk activity. Certain sectors of the population are more at risk of problem gambling and gambling harm. Consistent with most studies male gamblers are most at risk. In addition, young adults represent a new vulnerable cohort as they reach the legal age of gambling. This is true for most of the riskier activities except for racing which has an ageing cohort of gamblers.

In Victoria, there is a need to intervene earlier in the gambling journey to prevent and reduce harm to the population as well as the individual. Regulatory interventions, as well as the modification of structural characteristics of gambling products, are often effective options to reduce gambling harm.

## Data Availability

The data analysed during the current study were from Victorian surveys which were commissioned by the Department of Justice (2008), jointly by the Department of Justice and the Victorian Responsible Gambling Foundation (2014) and the Victorian Responsible Gambling Foundation (2018). Access to the data can be obtained by contacting the Victorian Responsible Gambling Foundation, Melbourne, Victoria, Australia.

## Footnotes

1 On EGMs, Casino EGMs and Table games, Wagering (racing, football, trackside and sports), lotteries and Keno.
